# How effective are ecological metrics in supporting conservation and management in degraded streams?

**DOI:** 10.1007/s10531-024-02933-7

**Published:** 2024-09-26

**Authors:** Kate L. Mathers, Christopher T. Robinson, Matthew Hill, Carmen Kowarik, Jani Heino, Charl Deacon, Christine Weber

**Affiliations:** 1 Geography and Environment, Loughborough University, Loughborough, Leicestershire, LE11 3TU UK; 2 Department of Surface Waters Research and Management, Eawag (Swiss Federal Institute of Aquatic Science and Technology), 6047 Kastanienbaum, Switzerland; 3 Department of Aquatic Ecology, Eawag, 8600 Dübendorf, Switzerland; 4 Institute of Integrative Biology, ETH Zürich, 8092 Zurich, Switzerland; 5 Department of Life and Environmental Sciences, Faculty of Science and Technology, Bournemouth University, Poole, Dorset, BH12 5BB UK; 6 Geography Research Unit, University of Oulu, P.O. Box 8000, Oulu, Finland; 7 Department of Conservation Ecology and Entomology, Stellenbosch University, Stellenbosch, South Africa

**Keywords:** Macroinvertebrates, Species identity, Environmental degradation, Heterogeneity, Environmental filtering, Richness

## Abstract

Biodiversity loss is increasing worldwide, necessitating effective approaches to counteract negative trends. Here, we assessed aquatic macroinvertebrate biodiversity in two river catchments in Switzerland; one significantly degraded and associated with urbanisation and instream barriers, and one in a near-natural condition. Contrary to our expectations, environmental heterogeneity was lower in the near-natural stream, with enhanced productivity in the degraded system resulting in a greater range of environmental conditions. At face value, commonly employed alpha, beta and gamma biodiversity metrics suggested both catchments constituted healthy systems, with greater richness or comparable values recorded in the degraded system relative to the near-natural one. Further, functional metrics considered to be early indicators for anthropogenic disturbance, demonstrated no anticipated differences between degraded and near-natural catchments. However, investigating the identity of the taxa unique to each river system showed that anthropogenic degradation led to replacement of specialist, sensitive species indicative of pristine rivers, by generalist, pollution tolerant species. These replacements reflect a major alteration in community composition in the degraded system compared with the near-natural system. Total nitrogen and fine sediment were important in distinguishing the respective communities. We urge caution in biodiversity studies that employ numerical biodiversity metrics alone. Assessing just one aspect of diversity, such as richness, is not sufficient to track biodiversity changes associated with environmental stress. We advocate that biodiversity monitoring for conservation and management purposes must go beyond traditional richness biodiversity metrics, to include indices that incorporate detailed nuances of biotic communities that relates to taxon identity.

**Supplementary Information:**

The online version contains supplementary material available at 10.1007/s10531-024-02933-7.

## Introduction

Novel and intensifying levels of anthropogenic pressures on ecosystems is leading to global and regional biodiversity declines at unprecedented rates, acting together with climate change (Sol et al. 2017; Cardoso et al. 2020). However, land use change and different forms of pollution remain equally important in conservation and management decisions (Maxwell et al. 2016; Titeux et al. 2016). At local and landscape scales, both pressures have been found to exert greater dominance in driving biodiversity losses than climate change in marine, terrestrial and freshwater ecosystems (Jaureguiberry et al. 2022). Rivers support a disproportionate level of biodiversity (Strayer and Dudgeon 2010), but have suffered losses far exceeding their terrestrial and marine counterparts (Grooten and Almond 2018; Tickner et al. 2020; Albert et al. 2021). Rivers often demonstrate elevated levels of anthropogenic modification due to high societal and ecosystem values (food, amenities, transport, energy), and high connectivity in the landscape (Lynch et al. 2016; 2023; Heino and Koljonen 2022). A recent call by Dudgeon (2019) stated that despite being under existential threat from climate change, freshwater systems are facing far greater concerns related to damming, habitat degradation and pollution.

The construction of instream barriers, such as dams and weirs, has led to hydrological and morphological impairment in many fluvial systems globally (Grill et al. 2019). In Europe alone, it is estimated that over 1.2 million barriers fragment fluvial networks (Belletti et al. 2020), which has multidimensional consequences regarding landscape connectivity and instream habitat quality, and with subsequent implications for food webs (Kondolf 1997; Wohl 2017). Together, changing land-use practices, and the increase in urban settlements and wastewater treatment discharges over the last century, have had significant effects on water quality and macronutrient levels (N and P) of freshwaters globally (Whelan et al. 2022; Heino et al. 2023). As such, habitat degradation via nutrient loads and fine sediment, which can act independently or in synergy, can have considerable effects on stream organisms (Wood and Armitage 1997; Matthaei et al. 2010; Robinson et al. 2014).

Environmental filtering suggests that biotic communities are organised along environmental gradients. Sensitive taxa are excluded as anthropogenic pressure increases, whereas generalist species are able to persist in sub-optimal and homogenised conditions. This, in turn, often leads to the homogenisation of biotic communities (Rolls et al. 2023), and a reduction in species diversity as the community becomes dominated by generalist species as multi-stress levels increase (Barnum et al. 2017). In this context, various alpha diversity metrics such as overall taxonomic richness and Ephemeroptera, Plecoptera and Trichoptera (EPT) richness form the basis of most biomonitoring programs as measures of lotic ecosystem health (Schmidt-Kloiber and Nijboer 2004; Callanan et al. 2008). Alongside gamma diversity (measured at landscape or catchment scale), these metrics may enable indication of whether species loss is occurring at a local scale (alpha richness) or whether there are coarser scale changes in taxa identity (Socolar et al. 2016).

However, some studies have found increasing alpha richness when anthropogenic disturbance leads to increasing resource levels and productivity (Hillebrand et al. 2018). As such, there may be incongruence between biodiversity metrics (Maloney et al., 2011), and numerical indices may overlook changes in species identity should specialist species be lost and replaced by generalist taxa. Application of different metrics in isolation or without consideration of species identity may therefore lead to misclassification of riverine ecosystem health and thus misguide conservation efforts.

In recent years, functional diversity based on biological traits has become a useful tool to evaluate biological patterns (Bonada et al. 2006; Schmera et al. 2017), including those associated with anthropogenic stressors (Mouillot et al. 2013; Barnum et al. 2017). Functional diversity offers a link between ecosystem functioning and biodiversity and is assumed to provide a proxy of mechanistic knowledge of why a change in biodiversity occurred rather than just observing the change itself. Trait-based community descriptors may represent a valuable biomonitoring tool and are particularly powerful when used together with traditional taxonomic metrics (Menezes et al. 2010; Paz et al. 2023). Measures of functional evenness, richness and divergence (Mouillot et al. 2013; Martins et al., 2021) are considered useful early warning tools for detecting riverine degradation. However, the relatively limited number of studies that have tested this assumption have shown conflicting results (Barnum et al. 2017; Ding et al. 2017), thus their use requires further consideration.

Alongside the increasing application of functional diversity indices, developments in beta diversity research (Anderson et al. 2011) also may enhance our ability to understand the effects of environmental stressors on freshwaters, with important practical applications for conservation efforts (Vilmi et al. 2017; Hill et al. 2021; Heino et al. 2022). Based on environmental filtering theory, beta diversity represents a potentially valuable metric to assess biodiversity when anthropogenic stress leads to biotic homogenisation (Rolls et al. 2023). A metric associated with beta diversity is called “local contribution to beta diversity” (LCBD; Legendre and De Cáceres 2013). This metric quantifies the ecological uniqueness of each site within a landscape context (Legendre and De Cáceres 2013; Heino and Grönroos 2017), and it can be further partitioned into replacement and richness difference components (Legendre 2014). In this context, understanding the processes affecting biodiversity can contribute to the development of conservation and management practices (Heino et al. 2023). High LCBD values may reflect unusual species composition and/or unique environmental conditions (high conservation value) or may represent degraded sites that support low taxa richness (high richness difference) that could be considered restoration targets (Legendre 2014). To date, the majority of LCBD studies were based on ecological data, but further insights into environmental processes structuring biodiversity associated with anthropogenic stressors may be gained by extending this approach to include environmental information (Castro et al. 2019; Heino et al. 2022).

In this study, we examined if a near-natural and a degraded lotic system differed in taxonomic and functional measures of macroinvertebrate communities. We hypothesised that: H1) alpha, beta and gamma diversity would be greatest in the near-natural system associated with greater habitat heterogeneity and good water quality, and H2) taxonomic and functional structure would be related to environmental conditions associated with the degree of anthropogenic disturbance present in each system. By using a combination of commonly employed community measures, we examined the processes structuring biodiversity, and tested whether all metrics provided effective biomonitoring and conservation tools.

## Material and methods

### Study systems

**Fig. 1:**
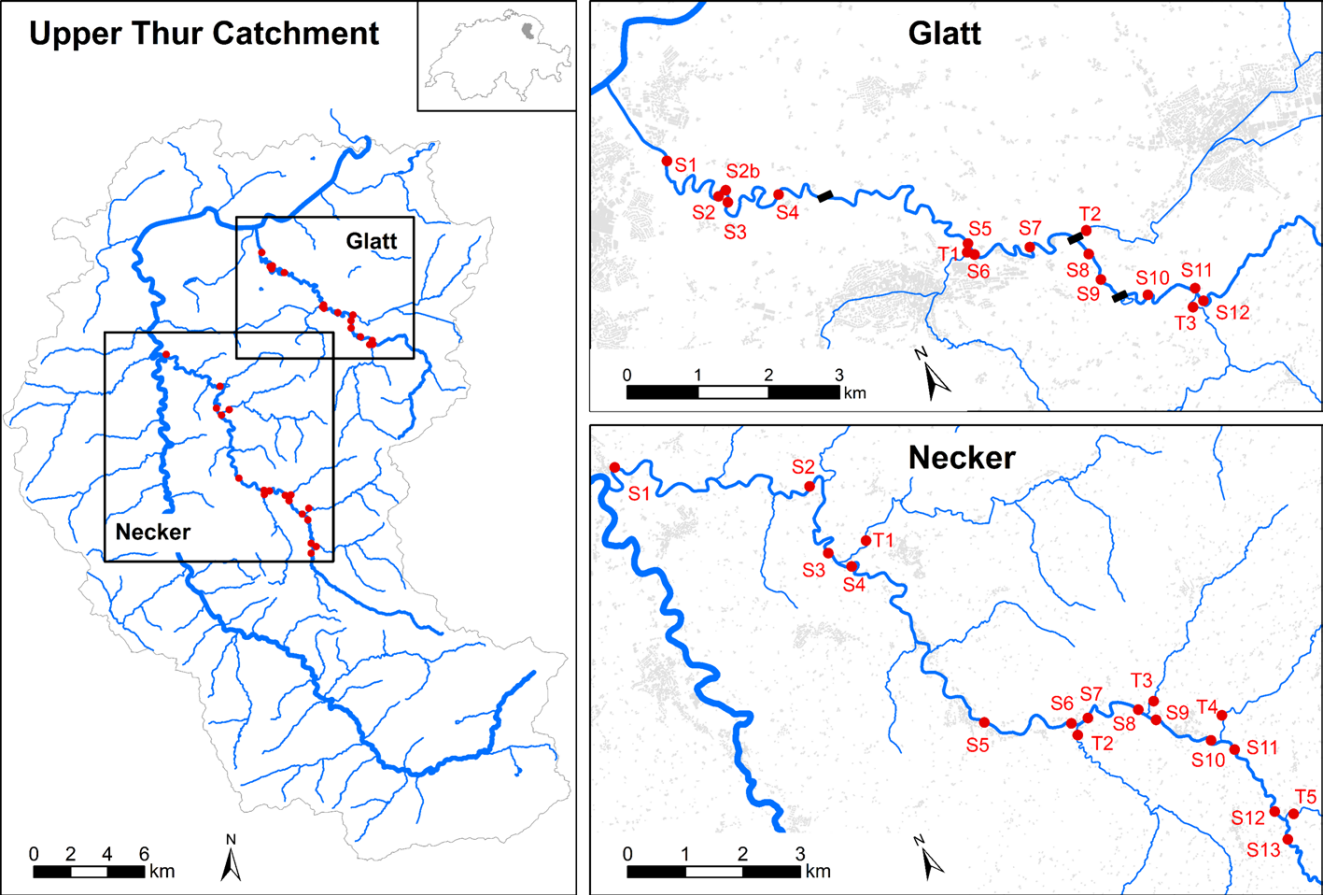
Map of the study site locations on the Glatt and Necker rivers in the Thur catchment (Switzerland)

The degraded River Glatt and near-natural River Necker, part of the North-Eastern pre-Alps, were selected for study (Fig. 1). Both 6th order rivers drain into the River Thur, a major tributary of the River Rhine. Near the catchment outlet, the Glatt has a mean annual flow of 2.3 m^3^ s^−1^ draining an area of 91 km^2^ while the Necker’s flow is 4.6 m^3^ s^−1^ across an area of 125 km^2^ (Federal Office of Topography, 2024). Carbonate sedimentary rocks dominate both catchments in a landscape consisting of a mix of mountain pastures replaced by forested and grassland in lowland areas. The urban area increases in frequency and size along the course of the two rivers, but is more pronounced in the Glatt catchment. The Glatt has also undergone significant anthropogenic modification that includes the construction of multiple barriers (three in the stretch we sampled), which has led to a 50–80% bed load deficit and morphological impairment downstream (Schälchli et al. 2005). The Glatt is predominantly a rain-fed system, whilst the Necker is a peri-alpine snow-fed system.

Sampling was conducted in autumn (October) along a ~ 10 km stretch of the main stems of both rivers with minimal elevation changes; 630–500 m asl, 0.5% slope, on the Glatt, 750–550 m asl, 0.7% slope, on the Necker (Federal Office of Topography, 2024). Thirteen sites were sampled on each river (Fig. 1) above and below the confluence of major tributaries as well as one site on the respective tributaries (three Glatt tributaries and five Necker tributaries). These main tributaries provide potential inputs of sediment as well as surface waters that can alter the physico-chemistry of waters in the main stem. In total, 34 sites were sampled across the two rivers (Glatt = 16, Necker = 18), representing a gradient of sediment conditions.

### Sampling methods

#### Sediment composition and channel stability

To assess the grain size distribution and variation of surface substrates, Wolman pebble counts, based on the measurement of 100 particles, were performed in one riffle and one pool at each sample site (n = 200/site; Wolman 1954). Sediment grains were selected along longitudinal transects within each habitat (pool or riffle), blindly selecting a grain at each step and measuring the b-axis via a gravelometer.

As Wolman counts can underestimate finer particles (Fripp and Diplas 1993), a resuspension technique using a stilling well (Lambert and Walling 1988; Collins and Walling 2007) was employed to quantify fine sediment content of surface and shallow subsurface (ca. 10 cm) areas of the river bottom. Sample patches were approached from downstream, and an open-ended plastic cylinder (height 42.5 cm, diameter 28 cm) was pushed manually into the riverbed until a seal with the substrate was achieved (i.e., no surface water flow evident through the cylinder). During this process, care was taken to minimize disturbance and prevent winnowing of fine material. Water depth in the cylinder was measured prior to sampling. First, the water within the cylinder was vigorously agitated using a spade for 60-s to suspend fine sediment on the riverbed surface into the water column. Following this period, a 50-ml vial was inverted in the water column, turned upright and capped as a surface fine-sediment sample. Subsequently, another 60-s of agitation was undertaken with a spade that included 30-s of disturbing the top 10 cm of the riverbed, and a second 50 ml sample then taken as total fine sediment content (interstitial and surface combined). Samples were taken from two erosional and two depositional areas to account for spatial variability in fine sediment deposition at each site (after Duerdoth et al. 2015). No water samples were taken because no major precipitation events preceded or occurred during the sampling period.

All suspended sediment samples were returned to the laboratory and refrigerated in the dark until further processing. Each sample was filtered using pre-ashed 0.45 μm Whatman glass microfiber filters, and oven dried at 105 °C overnight 1974 The water depth in the stilling well at each sample location was used to convert laboratory weights to a mass of fine sediment per square metre of riverbed for each surface and total fine sediment content sample.

Lastly, the Pfankuch stability score was calculated at each main-stem site (excluding tributaries). This score is a qualitative index that describes the degree of substrate-moving spates and the likelihood of substrate movement during high flows (Pfankuch 1975). The Pfankuch score involves the scoring of 15 parameters in three stream regions (upper banks, lower banks, and streambed), and has been successfully used to measure bank and streambed stability at a reach scale in association with aquatic macroinvertebrate research (Townsend et al. 1997; Li et al. 2019).

#### Hydraulic and water quality conditions

At each site, a minimum of five horizontal transect profiles were recorded consisting of five sample points at equidistant locations along the profile to ensure habitat variability was covered. Transects covered the range of habitats present, encompassing both slow and fast areas of flow velocity. For each sample point, water depth was measured (cm) and a 5 s averaged flow velocity (m/s) reading was collected at 40% from the riverbed using a Marsh McBirney Flo-Mate 2000. The wetted width (m) of the channel was measured for each of the five or more transect profiles.

At each site, electrical conductivity (μS cm^−1^), pH, water temperature (°C) and total dissolved solids (ppm) were measured using a Hanna Hi 9813–6 multiprobe. In addition, 500 ml water samples were collected from each sample site (all on one day) at the end of the sampling period and analysed for total nitrogen (TN, mg/L) and total phosphorus (TP, mg/L) in the laboratory following methods in Tockner et al. (1997).

#### Macroinvertebrate sampling

Macroinvertebrates were sampled from 18 to 23 October 2018. At each site, macroinvertebrates were sampled for 3 min using a kick technique with a standard kick net (500 μm mesh size). Sample time was divided equally between the different substrates (e.g., cobble, gravel) present at a site. Larger substrates (e.g., boulders) that could not be sampled with a pond net were visually/manually inspected for 60 s for macroinvertebrates. All samples were preserved in the field in 70% ethanol for subsequent identification in the laboratory. Macroinvertebrates were mostly identified to genus or species level with the exception of Hydracarina, Chironomidae, Simuliidae, Ceratopogonidae, Zonitidae and Collembola, which were recorded as such using the following keys; Studemann et al., (1992), Lubini et al., (2012), Friday (1988), Waringer and Graf (2011), Mauch (2017), Altermatt et al., (2019), Tachet et al. (2010) and Dobson et al. 2012.

#### Functional traits and habitat association

Macroinvertebrates were assigned to 11 biological ‘grouping features’ comprising 63 functional traits from the Tachet et al. (2010) European trait database (Table S1). Trait values were standardised following a ‘fuzzy coding’ standardisation (Chevene et al. 1994) using the ‘*prep.fuzzy’* function in the ade4 package (Thioulouse et al. 2018) so that each grouping feature summed to 1 (this ensures trait affinities had an equal weighting among taxa). Taxa recorded at a lower resolution than that of the trait database (e.g., species level) were aggregated, and for taxa recorded to a higher level than the database (e.g. family level) affinities of all recorded genera were averaged to provide a family score (sensu Gayraud et al. 2003). Functional traits were assigned to 88 taxa out of the 93 identified. Additionally, unique taxa for each river system were identified, and the biological preferences (as defined by Tachet et al. (2010)) of altitude, current velocity, trophic status and substrate were assigned to each taxon that uniquely occurred. As with the trait values, biological preferences were standardised (summed to 1) and the mean of all preference categories was calculated for each river. This provided us with a relative affinity for all unique taxa in each river system to better understand their habitat preferences.

#### Sedimentological and hydraulic conditions

Cumulative grain size distribution (GSD) curves were constructed from Wolman counts data from each site. Grain size percentiles of D16, D50, D84 and statistical parameters of mean, sorting, skewness, kurtosis (Bunte and Abt 2001) were derived to characterise substrate composition for each site. Substrate diversity was calculated from the substrate composition of each site by employing an ecologically meaningful spatial heterogeneity index; the Shannon’s diversity index (H). This metric has been successfully used as a measure of habitat heterogeneity in geomorphological and ecological studies (Yarnell et al., 2006; Turley et al. 2017) using the equation:

$$H = \Sigma pi \ln pi$$

where *pi* = the proportion of the streambed categorised as substrate size class *i*. Values were calculated using the *diverse* function in the vegan package in the R environment (Oksanen et al., 2022).

To assess hydraulic conditions, the Froude number was calculated for each flow velocity-depth transect point. The Froude number has been shown to be an ecologically meaningful parameter in determining habitat-scale distributions of macroinvertebrates (Lamouroux et al. 2004; White et al. 2019) and was calculated as:

$$Fr = v/\surd gD$$

where v = average velocity (ms^−1^), g = gravitational acceleration (ms^−2^), and D = water depth (m). To assess the heterogeneity in flow conditions, the coefficient of variation (CV; Wohl and Thompson 2000) in mean flow velocity for each sample site was calculated and used in subsequent analysis.

### Statistical analysis

All statistical analyses were undertaken in R version 4.1.1 (R Development Core team, 2021).

#### Environmental variables

Differences in physico-chemical conditions between the two rivers (including tributaries) were examined via Principal Component Analyses (PCA) using the ‘*prcomp*’ function in the ‘stats’ package. Highly correlated variables (Pearson’s *r’*s > 0.75) were considered redundant, and only the most biologically relevant variables were retained to minimize collinearity. The final 8 variables (reduced from the 21 measured) included in the model were pH, total phosphorus, total nitrogen, D50, D84, Shannon substrate, Froude CV, and total fine sediment. Statistical significance of individual environmental parameters associated with the two rivers were examined using a Mann–Whitney U test with the ‘*wilcox.test’* function in R. As the Pfankuch index was recorded only for main stem sites, index scores were assessed using the Mann–Whitney U test only and not within the PCA.

A permutational multivariate analysis of variance (PERMANOVA; 999 permutations) was undertaken to test for differences in the environmental template between rivers using the ‘*adonis*’ function in the vegan package of R (Oksanen et al., 2022). Non-Metric Multidimensional Scaling (NMDS), with the ‘*metaMDS’* function in vegan, was used to graphically display the heterogeneity of environmental conditions (using standardised Euclidean distances) between rivers. The homogeneity of multivariate dispersions (PERMDISP) was calculated using the ‘*betadisper*’ function in vegan with differences tested using Analysis of Variance (ANOVA). Additionally, the local contribution to environmental heterogeneity was calculated (LCEH), which quantifies the environmental uniqueness of each site within the context of the full dataset, with high values for a given site indicative of high environmental dissimilarity to other sites. A site-by-site environmental matrix was calculated using Euclidean distances before calculating LCEH (Castro et al. 2019; Heino et al. 2022). The ‘*LCBD.comp’* function in the adespatial package (Dray et al. 2023) was used on the environmental distance matrix to derive local contributions to environmental heterogeneity and statistically tested using a Mann–Whitney U test as described above. All environmental characteristics were standardised (each parameter had a zero mean and unit variance) prior to the environmental NMDS, PERMANOVA, betadisper and calculation of LCEH.

#### Gamma and alpha diversity

Gamma diversity reflected the total diversity present in each river (Glatt, Necker) and alpha diversity the number of macroinvertebrate taxa present at each study site. Gamma diversity was calculated with the Chao estimator using the ‘*specpool*’ function in vegan, which uses the number of uncommonly occurring taxa in a sample to estimate the number of undiscovered species (Oksanen et al. 2019). Differences in gamma diversity between rivers were considered significant if the 95% confidence intervals did not overlap.

Six alpha diversity metrics were calculated, including three taxonomic metrics (community abundance, taxa richness and Ephemeroptera, Plecoptera and Trichoptera (EPT) richness) and three functional diversity metrics (functional richness (FRic) representing the minimum convex hull encompassing all species, functional evenness (FEve) reflecting the regularity in which species are distributed across functional space, and functional divergence (FDiv) representing how abundance is distributed within the volume of functional space occupied by species). The functional diversity metrics were computed using the ‘*dbFD’* function on a Gower distance matrix in the FD package (Laliberté, et al., 2014). Differences in alpha diversity measures between rivers were determined using Mann–Whitney U tests.

#### Macroinvertebrate community composition

Beta-diversity is defined here as the variation between community assemblages within a predefined geographical area (after Whittaker 1960). Prior to all functional beta diversity analyses, the dimensionality of the taxa-by-traits matrix was reduced using the Gower distance to provide a taxa-by-taxa functional distance matrix (‘*gowdis’* function in the FD package), which underwent hierarchical clustering using an unweighted pair group method with arithmetic means (UMPGA in phangorn package; Schliep, 2011). Thereafter, following Cardoso et al. (2015), the resulting functional tree was analysed along with sites-by-taxa matrix to provide functional site-by-site dissimilarity matrix using the *beta* function in the BAT package. As with the environmental data, beta diversity using taxonomic and functional compositional differences between rivers was visually examined via NMDS (using the Jaccard dissimilarity metric for taxonomic communities and the functional dissimilarity matrix calculated above), with differences in community composition (taxonomic and functional) between rivers tested via PERMANOVA. Differences in homogeneity of multivariate dispersions between the two rivers were calculated using the PERMDISP method with the ‘*betadisper’* function and tested using ANOVA.

Beta-diversity, and the contribution of the replacement and richness difference components, were calculated to investigate the dominant processes structuring taxonomic and functional differences in macroinvertebrate composition among study sites, and the Glatt and Necker independently. For taxonomic data, the ‘*beta.div.co*mp’ function in the adespatial package was employed using the Podani family with Jaccard-based indices. Functional beta-diversity, species replacement, and richness difference pairwise distance matrices were calculated using the ‘*beta*’ function in the BAT package (Cardoso et al. 2024; 2020).

Lastly, the Local Contribution to Beta Diversity (LCBD) was calculated from the taxonomic data (Legendre and Caceres, 2013). Taxonomic LCBD values (LCBD) were calculated using the Jaccard dissimilarity metric (on presence/absence data) via the function ‘*beta.div’* in the adespatial package. The significance of individual LCBD values were calculated through random, independent permutations within the community matrix (Legendre and Cáceres 2013). The ‘*beta.div.comp’* function (Podani family) in the adespatial package was used to calculate the contribution of species replacement (LCBD_repl_) and richness difference (LCBD_richdiff_) to individual site LCBD values. For functional community data, the derived beta-diversity pairwise dissimilarity matrix outlined above was used to determine functional LCBD (LCBD-f) values for each community using the *‘beta.div’* function in the adespatial package (Heino et al. 2022). Differences in LCBD, LCBD_repl_, LCBD_richdiff_, and LCBD-f between rivers were tested using Mann Whitney U tests.

#### Species-environment and trait-environment relationships

Following the PCA analysis, the same eight environmental variables were used in redundancy analysis (RDA) to examine the relationship between the taxonomic macroinvertebrate community and environmental variables. Final variables were checked for collinearity using the *vif* function in the car package to ensure that all ‘variance inflation factors’ were < 3 (Zuur et al. 2010). Prior to analysis, potential within-river spatial autocorrelation in both taxonomic and functional community dissimilarity was examined using a Mantel test (Mantel 1967) via the ‘*mantel’* function in vegan based on the Pearson correlation coefficient and 999 permutations. To conduct the RDA analysis, a Hellinger transformation (Legendre and Gallagher 2001) was first applied to the species-abundance data, and distance-based redundancy analysis (db-RDA) was undertaken for the functional community data (Perez Rocha et al. 2018). The functional total pairwise dissimilarity matrix was used as input data in the latter. A db‐RDA was run using the *capscale* function in vegan, with the sqrt.dist correction for negative eigenvalues.

Significant environmental variables influencing functional and taxonomic measures of macroinvertebrate communities were identified using the *ordiR2step* function, and adjusted r^2^ values calculated using the *RsquareAdj* function in vegan. The significance of the full taxonomic RDA and functional db-RDA models was tested using ANOVA. Subsequently, partial taxonomic RDA and functional db-RDA analyses were run to identify the individual contribution of significant environmental variables to the variation in taxonomic and functional macroinvertebrate community composition (Borcard et al. 1992). Significant environmental variables in the RDA models were treated as explanatory variables, and the other environmental variables were considered covariates to obtain the individual effect of each variable using ANOVA.

## Results

### Environmental template

**Fig. 2:**
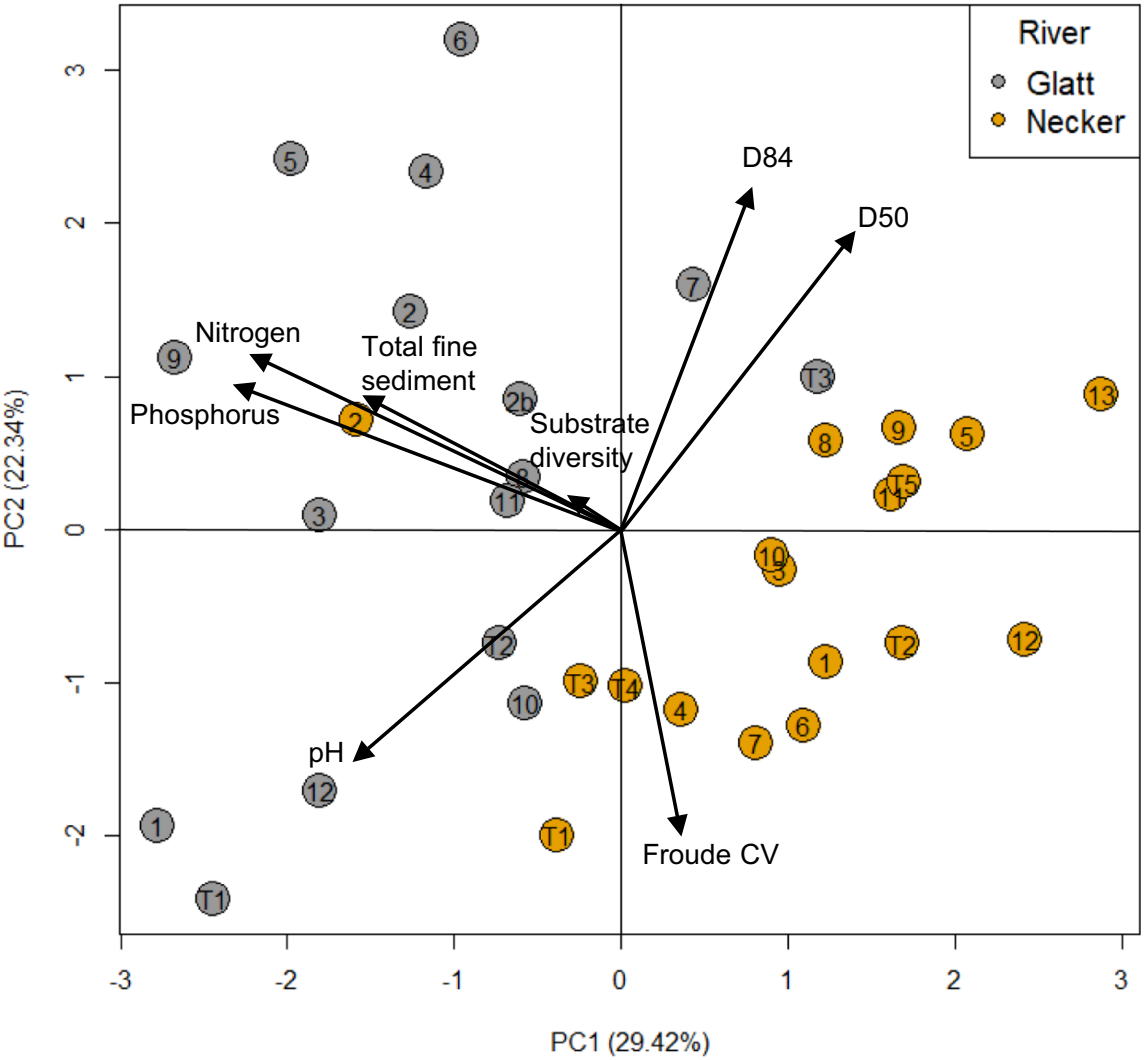
Principal component analysis plots of physico-chemical data from the Glatt and Necker rivers. Note the Pfankuch index is not included in this analysis as it was quantified only for main stem sites on each river. Numbers represent site locations along each river

The PCA indicated differences in the physico-chemistry between rivers, and mostly on PC1 (explaining 29.4% of the variance). All but two Glatt sites plotted negatively on PC1 along with total nitrogen, total phosphorus, total fine sediment and pH (Fig. 2). All variables exhibited significant variability, except for Shannon substrate diversity that displayed low loadings on both PC1 and PC2. Neither the Glatt nor Necker demonstrated a longitudinal pattern in the environmental template (Fig. 2). Levels of total phosphorus (W = 271, p < 0.001), total nitrogen (W = 286.5, p < 0.001) and total fine sediment (W = 244, p < 0.001) were statistically higher in the Glatt (Figures S1a-c), whilst pH (W = 199.5, p = 0.056), D50 (W = 124, p = 0.51) and D85 (W = 186, p = 0.82) did not vary between rivers. Environmental conditions differed between rivers (PERMANOVA R^2^ = 0.16, p < 0.001; Figure S2), with environmental multivariate dispersion being greater in the Glatt (average distance to centroid: 0.29) than the Necker (distance: 0.24; Pairwise ANOVA, F = 6.30, p = 0.017). Moreover, LCEH was highest in the Glatt (W = 202, p = 0.046; Figure S3). The Glatt sites with the greatest environmental uniqueness were G1, G2, G5, G9 and GT1. The Pfankuch index was greater in the Glatt than the Necker (W = 130, p = 0.021; Figure S1d).

### Gamma and alpha diversity

In both rivers, 93 macroinvertebrate taxa and 31,611 individuals were recorded from 19 orders and 60 families. The most widely recorded taxa were Chironomidae (34 sites), *Baetis* sp. (34 sites), Elmidae (34 sites), *Hydropsyche* sp. (33 sites), *Rhyacophila* sp. (33 sites), *Rhithrogena* sp. (32 river sites), *Ecdyonurus* sp. (32 river sites) and Simuliidae (32 sites). The Glatt had a higher macroinvertebrate richness (79 taxa) and abundance (16,654 individuals) than the Necker (69 taxa and 14,957 individuals; Figure S4). However, no significant difference (p > 0.05) was found in gamma diversity between the Glatt (gamma: 114.2, 95% CI: 76.9–151.5) and the Necker (gamma: 87.9, 95% CI: 65.2–110.6).

**Fig. 3:**
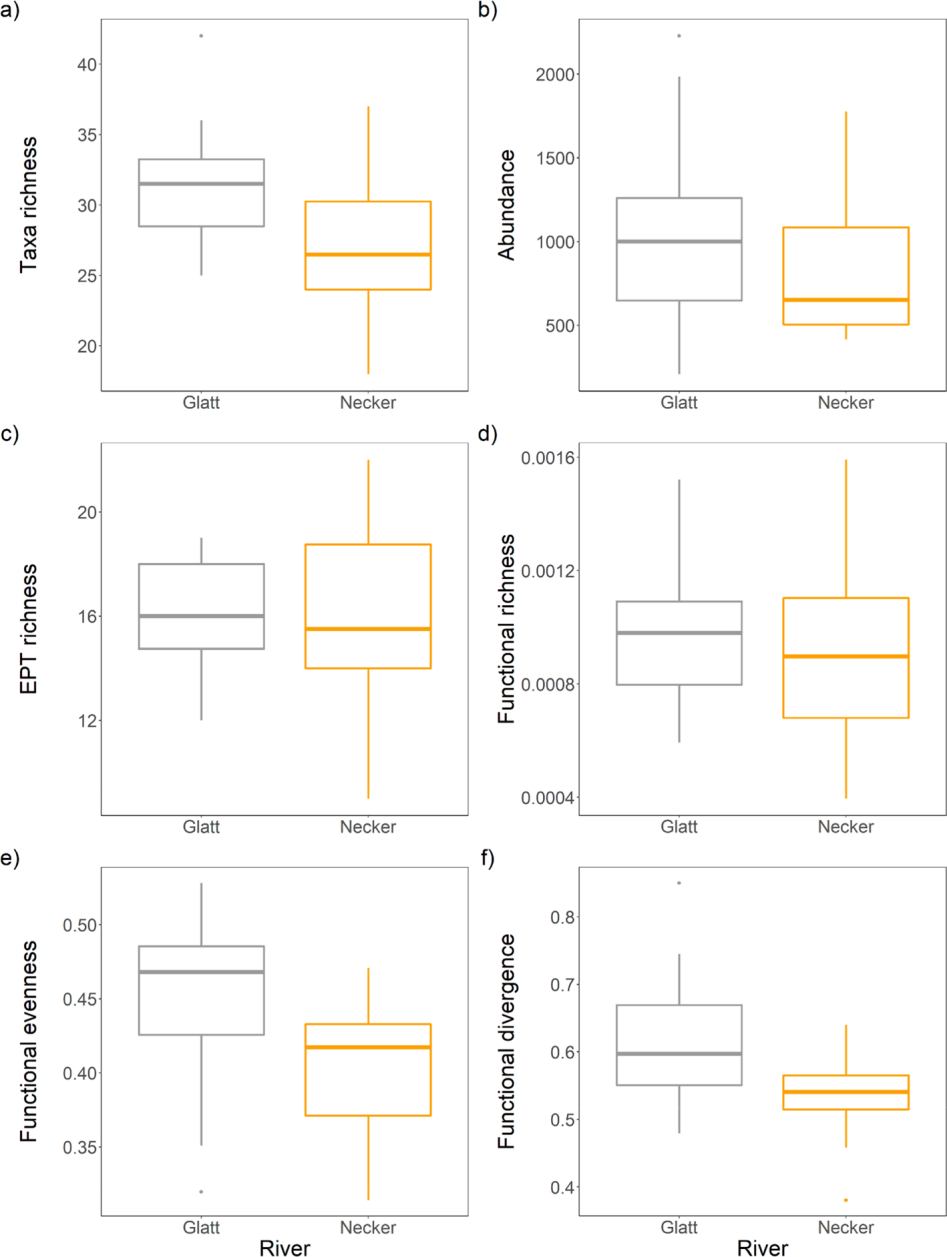
Mean (± 1 SE). **a** taxa richness, **b** abundance, **c** EPT richness, **d** functional richness, **e** functional evenness, and **f** functional divergence for the Glatt and Necker rivers

For alpha diversity, the Glatt had greater macroinvertebrate richness compared to the Necker (W = 211, p = 0.021; Fig. 3a). No differences were recorded between rivers in abundance (W = 174, p = 0.31), EPT richness (W = 147.5, p = 0.92) and functional richness (W = 167, p = 0.44; Fig. 3b-d). However, the Glatt had greater values of functional evenness (W = 217, p = 0.011) and functional divergence (W = 211, p = 0.020; Figs. 3e & f) than the Necker.

### Macroinvertebrate community composition

**Fig. 4:**
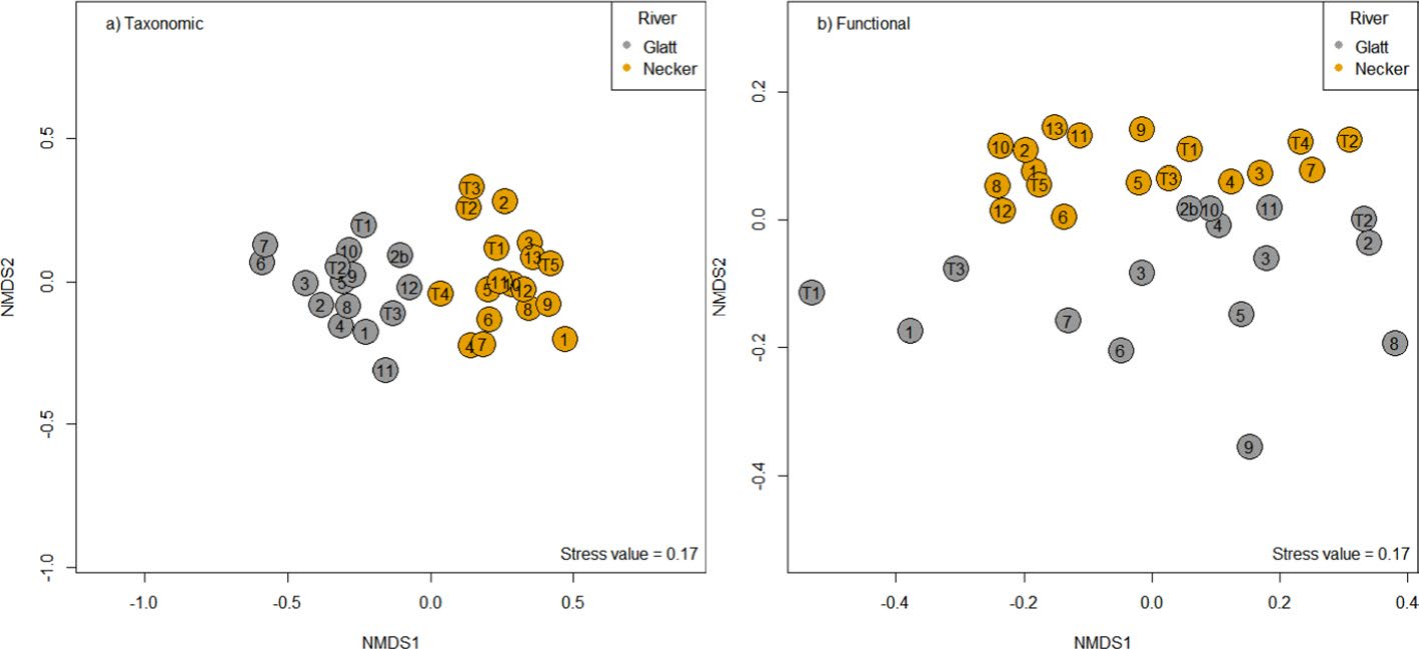
Non-metric multidimensional scaling (NMDS) of **a** taxonomic and **b** functional macroinvertebrate communities of the Glatt (grey) and Necker (orange) rivers. Numbers represent the site locations along each river

Both taxonomic (PERMANOVA R^2^ = 0.21, p < 0.001) and functional (PERMANOVA R^2^ = 0.124, p = 0.002) macroinvertebrate community composition differed between rivers (Fig. 4a, b). Neither the Glatt nor Necker demonstrated a longitudinal pattern in the macroinvertebrate communities (Fig. 4). No statistical differences (p > 0.05) in multivariate dispersion were found between macroinvertebrate communities in the Glatt (average distance to centroid: taxonomic: 0.38, functional: 0.16) and Necker (average distance to centroid: taxonomic: 0.33; functional: 0.14), although greater variability in taxonomic multivariate dispersion was evident in the Glatt (Figure S5).

Both rivers had relatively low total taxonomic beta-diversity (Glatt: 0.23, Necker: 0.23 using Jaccard metrics). Most variation in community composition was associated with species replacement rather than richness differences when both rivers were considered together (species replacement: 72%), and separately for the Glatt (species replacement: 75%) and Necker (species replacement: 62%). A moderate average community dissimilarity was found between the Glatt and Necker (average pairwise total beta-diversityLCB: 0.61). A total of 75% of the macroinvertebrate community dissimilarity between the Glatt and Necker was associated with species replacement (average pairwise species replacement: 0.46), while 25% was driven by richness differences (average pairwise richness difference: 0.15).

Functional macroinvertebrate composition showed a moderate value of total beta-diversity for the Glatt (0.61) and Necker (0.53). Richness differences structured most of the functional variation when all sites were combined (richness difference: 69%), and separately for the Glatt (richness difference: 75%) and Necker (richness difference: 64%). A moderate community dissimilarity was found (average pairwise total beta-diversity: 0.63) in functional community composition between rivers. A total of 67% of the macroinvertebrate community dissimilarity between the Glatt and Necker was attributed to richness differences (average pairwise richness difference: 0.42), while 33% was due to species replacement (average pairwise species replacement: 0.21).

A total of 23 macroinvertebrate taxa were unique to the Glatt and 14 taxa to the Necker, while 54 taxa were present in both rivers (Table 1). Of the taxa unique to the Glatt, eight taxa were Mollusca, five Diptera, three Trichoptera, two Crustacea, one Zygoptera, one Hirudinea, one Odonata, one Coleoptera and one Plecoptera. In contrast, taxa unique to the Necker comprised highly sensitive Plecoptera (e.g., *Dinocras cephalotes*, *Isoperla grammatica*, *Perla grandis*, *Perla marginata*), four Coleoptera including one endangered National red list species (*Oreodytes septentrionalis*; BUWAL 1994), two Trichoptera, two Hemiptera, and two Diptera. The overall biological preferences of the unique taxa in each river system indicated highly sensitive taxa that preferred alpine / piedmont river systems, fast flowing current velocities, oligotrophic water quality and gravel substrates in the Necker system (Figure S6). In marked contrast, unique taxa in the Glatt represented those that were recorded more often in lowland rivers, null / slow current velocity, tolerated eutrophic waters and preferred mud substrates (Figure S6).

**Table 1:** Aquatic macroinvertebrate taxa recorded that were unique to the River Glatt or River Necker

Unique to Glatt	Unique to Necker
*Nemoura* sp.	*Perla grandis*
Halipidae larvae	*Perla marginata*
*Onychogomphus* sp.	*Dinocras cephalotes*
*Calopteryx* sp.	*Isoperla grammatica*
*Astacus astacus*	Curculionidae
*Asellus aquaticus*	Scirtidae larvae (cf. *Elodes* sp*.*)
*Hyporhyacophila* sp.	*Oreodytes sanmarkii*
*Silo nigricornis*	*Oreodytes septentrionalis*
Leptoceridae (1st)	*Odontocerum albicorne*
Erpobdellidae	Philopotamidae (cf. *Philopotamus* sp*.*)
*Tabaninae* sp.	*Pedicia* sp.
Tabanidae (other)	*Limnophila* sp.
*Clinocera nigra*	*Microvelia* sp.
Ephydridae	*Mesovelia* sp.
*Stratiomys sp.*	
*Sphaerium* sp.	
*Psidium* sp.	
*Potamopyrgus antipodarum*	
*Physella acuta*	
*Physa fontinalis*	
*Planorbis albus*	
*Hippeutis complanatus*	
*Ancylus fluviatilis*	

Across the entire dataset, two sites recorded significant (p < 0.05) taxonomic LCBD values (Glatt: G6, G7). No differences in LCBD (W = 157, p = 0.67), LCBD_repl_ (W = 188, p = 0.13) and LCBD_richdiff_ (W = 96, p = 0.1) values were found between sites in the Glatt and Necker. However, greater variability in LCBD values was found for the Glatt compared to the Necker. No sites recorded significant functional LCBD values and no differences in LCBD-f were found between the Glatt (median: 0.029) and Necker (median: 0.031, W = 140, p = 0.905).

### Species-environment and trait-environment relationships

**Fig. 5:**
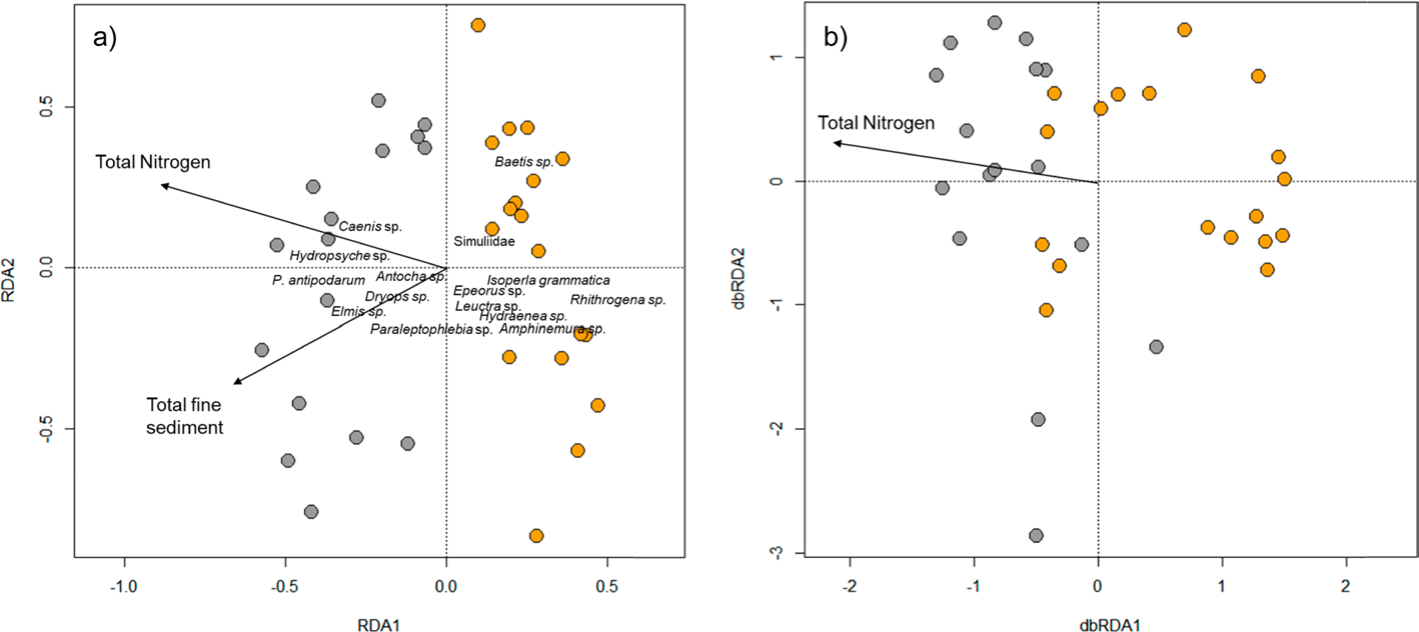
**a** Redundancy analysis on taxonomic macroinvertebrate communities, and **b** distance-based redundancy analysis on functional macroinvertebrate communities. Data collected from sites on the Glatt and Necker rivers in Switzerland. Only significant environmental parameters are shown. River Glatt = grey and River Necker = orange. Note, specific functional traits cannot be labelled on the functional plot due to the method used

Mantel correlation tests showed that Euclidean distances of taxonomic and functional communities in the Glatt (taxonomic r = -0.13, p = 0.923, functional r = -0.14, p = 0.943) and Necker (taxonomic r = r = -0.03, p = 0.561, functional r = -0.21, p = 0.065) demonstrated no within-river spatial autocorrelation. Taxonomic redundancy analysis showed that the Glatt and Necker macroinvertebrate communities were separated on the first and second axes along gradients associated with total nitrogen and total sediment (both p < 0.05, Fig. 5a). The RDA model was significant (F = 2.45 p = 0.001), explaining 28% of the variation in macroinvertebrate community composition on all axes. Sites in the Glatt were associated with greater total nitrogen and total fine sediment than sites in the Necker (Fig. 5a). Partial RDA indicated that total nitrogen contributed 14% (p = 0.001) and total sediment 4% (p = 0.027) to the total compositional variation in communities.

Results of db-RDA for functional community data also showed that the Glatt and Necker sites were separated on axis-1 along a gradient associated with total nitrogen (F = 2.70, p = 0.002, Fig. 5b). The db-RDA model was significant (F = 1.23, p = 0.049), explaining 5.2% of the variation in macroinvertebrate community composition. Partial db-RDA indicated that total nitrogen contributed 4.1% (p = 0.05) to the total compositional variation in communities.

## Discussion

In this study, we examined how anthropogenic degradation affects alpha, beta and gamma diversity of stream aquatic invertebrates. In contrast to our hypothesis (H1), we observed that alpha diversity (taxa richness) was greater in the degraded than near-natural river, whilst community abundance, EPT richness (widely used as a bioindicator of pollution-sensitive taxa including fine sediment deposition) and functional richness demonstrated no statistical differences between rivers. Similarly, gamma and beta diversity were comparable between the two rivers, and only the Glatt recorded significant LCBD values (two sites). However, the two rivers supported considerably different taxonomic and functional compositions. Differences between rivers in structural composition of invertebrate communities were due to species replacement (75%), whilst functional community composition differences were driven by richness differences (67%). LCBD showed no significant differences between rivers, most likely reflecting the relatively low heterogeneity between macroinvertebrate communities in the rivers. No significant variability in environmental conditions were evident for the Necker, whereas environmental conditions in the Glatt were highly variable with significant heterogeneity and Glatt sites demonstrated greater environmental uniqueness based on LCEH values than Necker sites. Despite this, no significant differences were recorded in multivariate dispersion of invertebrate communities between the rivers. This finding suggests that despite environmental conditions being highly heterogeneous in the Glatt, associated with different environmental stressors (total nitrogen and fine sediment), the general degradation of the system (habitat quality) has led to considerable environmental filtering resulting in a relatively homogeneous community throughout. The pool of taxa present in the Glatt consisted of generalist, highly tolerant taxa that occur throughout most of the system (Gafner and Robinson 2007).

In the Glatt, anthropogenic stress has increased environmental extremes throughout the system and more heterogeneous abiotic conditions, (based on greater environmental variability in multivariate dispersion), suggesting that the system is in an ongoing state of anthropogenic disturbance. This is contrast to the anticipated homogenisation of environmental conditions in the degraded system. We also observed that there was no longitudinal pattern in the environmental template or macroinvertebrate communities in either river (as shown in the multivariate plots). It appears therefore that the higher levels of resources via nutrient enrichment in the Glatt is supporting a comparably more diverse species pool than the Necker, even though species composition and community structure has been altered. The dynamic equilibrium model (Huston 1979, 1994), which integrates the intermediate disturbance (Connell 1978) and intermediate productivity (Grime 1973) hypotheses, highlights the interlinked nature of disturbance and productivity (e.g., resources). Despite the morphological impairment and longitudinal/lateral disconnection resulting from instream barriers, degradation in the system is moderately enhancing productivity, and suitable habitat for macroinvertebrates is still present for disturbance-tolerant species at the expense of ecologically sensitive ones (following Ward et al. 1999).

We tested two common functional metrics as indicators of environmental stress. Functional divergence is suggested to act as an early warning indicator of environment stress, as functional traits that are more sensitive to land use disturbance will typically lie on the fringe of trait space and thus are the first to be lost, resulting in reduced functional divergence values (Mouillot et al. 2013; Martins et al., 2021). However, we found that the degraded Glatt had greater functional divergence values than the near-natural Necker. The findings of Barnum et al. (2017) were similar; increasing urbanisation at the ecoregion level led to increasing functional divergence values. They suggested that functional divergence is not an early warning signal as hypothesised but still provides mechanistic insight into the redistribution of trait combinations in functional trait space. It is likely that the environmental implications of anthropogenic disturbance determine the ecological consequences for instream communities due to physical habitat alterations (flow, substrate quality, morphology), resulting in different ecological consequences than simply enhanced nutrient concentrations (Wagenhoff et al. 2011).

Low functional divergence values suggest that resource efficiency is low (Mason et al., 2005). However, productivity was much higher in the degraded Glatt than the near-natural Necker; e.g., nitrogen and phosphorus levels were greater in the Glatt. Periphyton biomass also was an order of magnitude greater in the Glatt (Glatt = 1.2 × 10^–3^ g cm^−3^, Necker = 2.4 × 10^–4^ g cm^−3^; n = 30/river; Kowarik unpublished data). This result is mirrored by the greater values of functional divergence and evenness observed in the degraded Glatt. Low functional evenness (as observed in the Necker) suggests that although some parts of the trait niche space are occupied, these are being underutilised. In contrast, increased functional divergence in the degraded system likely reflects the increased productivity leading to greater niche space being available, which is evenly occupied. Similar findings of greater functional evenness were observed as primary productivity increases, being associated with increasing nutrient levels (Rideout et al. 2022). Results from the trait-environment and species-environment analyses provide further support with total nitrogen levels significantly influencing the structure of both taxonomic and functional communities (H2). Although functional metrics provide mechanistic evidence of underlying ecosystem processes driving community structure, they may not provide a generalizable indicator of environmental stress without contextual knowledge.

### Importance of taxonomic identity for applied conservation

Our study demonstrates the importance of taxonomic knowledge of the taxa inhabiting a study system. By investigating community measures alone, it may appear that the degraded Glatt supports potentially more ecologically diverse taxa or that there is limited change in the richness of the perceived indictor group of EPT. Taking these result at face value could suggest that no conservation action is needed, or lead to misguided and ineffective conservation management strategies, particularly for the sensitive pre-Alpine streams investigated here and which support some species of conservation concern. Assessing just one aspect of diversity (e.g., richness or abundance) is insufficient to track biodiversity change associated with environmental stress, as reductions in environmental quality can often lead to increases in species richness (Hillebrand et al. 2018). Here, we emphasise that one should avoid attaching only one numerical value to diversity, but that a combination of biotic metrics and an underpinning knowledge of taxa identity are required to comprehensively understand the processes (such as anthropogenic degradation) structuring biodiversity, particularly when considered in the context of species turnover and replacement (Hillebrand et al. 2018; Li et al. 2020).

Our findings emphasise the strong limitation of employing biotic metrics without taxonomic knowledge of the species present in the systems being studied. Without considering the taxonomic identity of species, the value in fundamental diversity can be lost. Many of the unique taxa occurring in the Necker represent highly sensitive species, indicative of pristine or near-natural rivers that prefer fast current velocities, alpine or piedmont altitudes and clean waters. These specialist taxa were lost and replaced by generalists with stream degradation in the Glatt. Indeed, taxa unique to the Glatt were pollution-tolerant generalist species that prefer mud substrata, standing water or slow flows and are representative of lowland rivers. These include various Diptera and a large number of Mollusca, including the non-native mud-snail (*Potamopyrgus antipodarum*)*.* Several other studies have recorded similar views that specialist sensitive taxa are being replaced by generalist taxa, and that purely numerical richness metrics fail to detect this fundamental shift in community identity (Larsen et al. 2018; Hilpold et al. 2018).

Contemporary ecologists often have limited training in taxonomic methods and thus are unfamiliar with the natural histories and taxonomic knowledge of the organisms within their datasets/study sites. Such qualitative information is, however, vital for better mechanistic understanding of underlying ecological processes and patterns, which can better inform conservation practice (Kim and Byrne, 2006). However, taxonomic and trait information on species in mega-diverse and/or unexplored areas is often sparse, challenging the ability to incorporate these layers of information into conservation decisions. In these cases, numerical analyses of biodiversity patterns are useful, yet we urge ecologists to develop skills in taxonomic methods to gain better understanding of underlying bio-assembly rules. Like our findings, a number of community ecology studies have found that taxa identity and their natural histories are more important for determining community structure than species richness patterns, and thus tracking species identity is essential (Olden and Rooney, 2006). Greater integration of taxonomy and ecology must be a priority for ecological and applied biodiversity studies moving forward (Gotelli, 2004; Hillebrand et al. 2018).

## Supplementary Information

Below is the link to the electronic supplementary material.

Supplementary file1 (PPTX 7247 KB)

## Data Availability

Data are available from the corresponding author on reasonable request.
