# Supplementary material for: How effective are ecological metrics in supporting conservation and management in degraded streams?
Source: Biodivers Conserv. 2024 Sep 26;33(14):3981–4002. doi: 10.1007/s10531-024-02933-7 (PMC11568992; doi:10.1007/s10531-024-02933-7)
Supplement: Supplementary file 1 — Supplementary file1 (PPTX 7247 KB) [file 10531_2024_2933_MOESM1_ESM.pptx]

## Slide 1
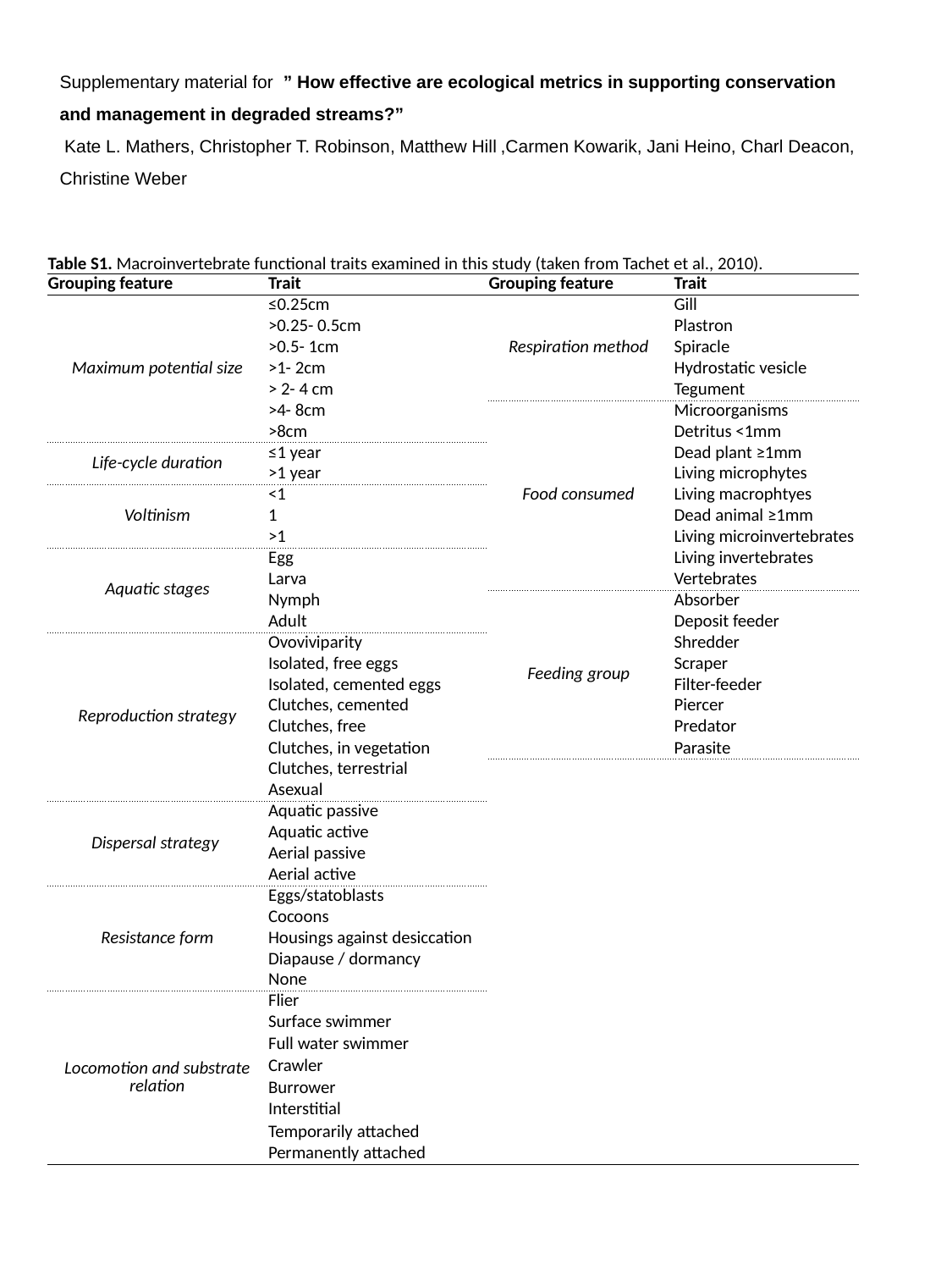

# Supplementary material for  ” How effective are ecological metrics in supporting conservation and management in degraded streams?”  Kate L. Mathers, Christopher T. Robinson, Matthew Hill ,Carmen Kowarik, Jani Heino, Charl Deacon, Christine Weber
| Table S1. Macroinvertebrate functional traits examined in this study (taken from Tachet et al., 2010). | | | |
| --- | --- | --- | --- |
| Grouping feature | Trait | Grouping feature | Trait |
| Maximum potential size | ≤0.25cm | Respiration method | Gill |
| | >0.25- 0.5cm | | Plastron |
| | >0.5- 1cm | | Spiracle |
| | >1- 2cm | | Hydrostatic vesicle |
| | > 2- 4 cm | | Tegument |
| | >4- 8cm | Food consumed | Microorganisms |
| | >8cm | | Detritus <1mm |
| Life-cycle duration | ≤1 year | | Dead plant ≥1mm |
| | >1 year | | Living microphytes |
| Voltinism | <1 | | Living macrophtyes |
| | 1 | | Dead animal ≥1mm |
| | >1 | | Living microinvertebrates |
| Aquatic stages | Egg | | Living invertebrates |
| | Larva | | Vertebrates |
| | Nymph | Feeding group | Absorber |
| | Adult | | Deposit feeder |
| Reproduction strategy | Ovoviviparity | | Shredder |
| | Isolated, free eggs | | Scraper |
| | Isolated, cemented eggs | | Filter-feeder |
| | Clutches, cemented | | Piercer |
| | Clutches, free | | Predator |
| | Clutches, in vegetation | | Parasite |
| | Clutches, terrestrial | | |
| | Asexual | | |
| Dispersal strategy | Aquatic passive | | |
| | Aquatic active | | |
| | Aerial passive | | |
| | Aerial active | | |
| Resistance form | Eggs/statoblasts | | |
| | Cocoons | | |
| | Housings against desiccation | | |
| | Diapause / dormancy | | |
| | None | | |
| Locomotion and substrate relation | Flier | | |
| | Surface swimmer | | |
| | Full water swimmer | | |
| | Crawler | | |
| | Burrower | | |
| | Interstitial | | |
| | Temporarily attached | | |
| | Permanently attached | | |

## Slide 2
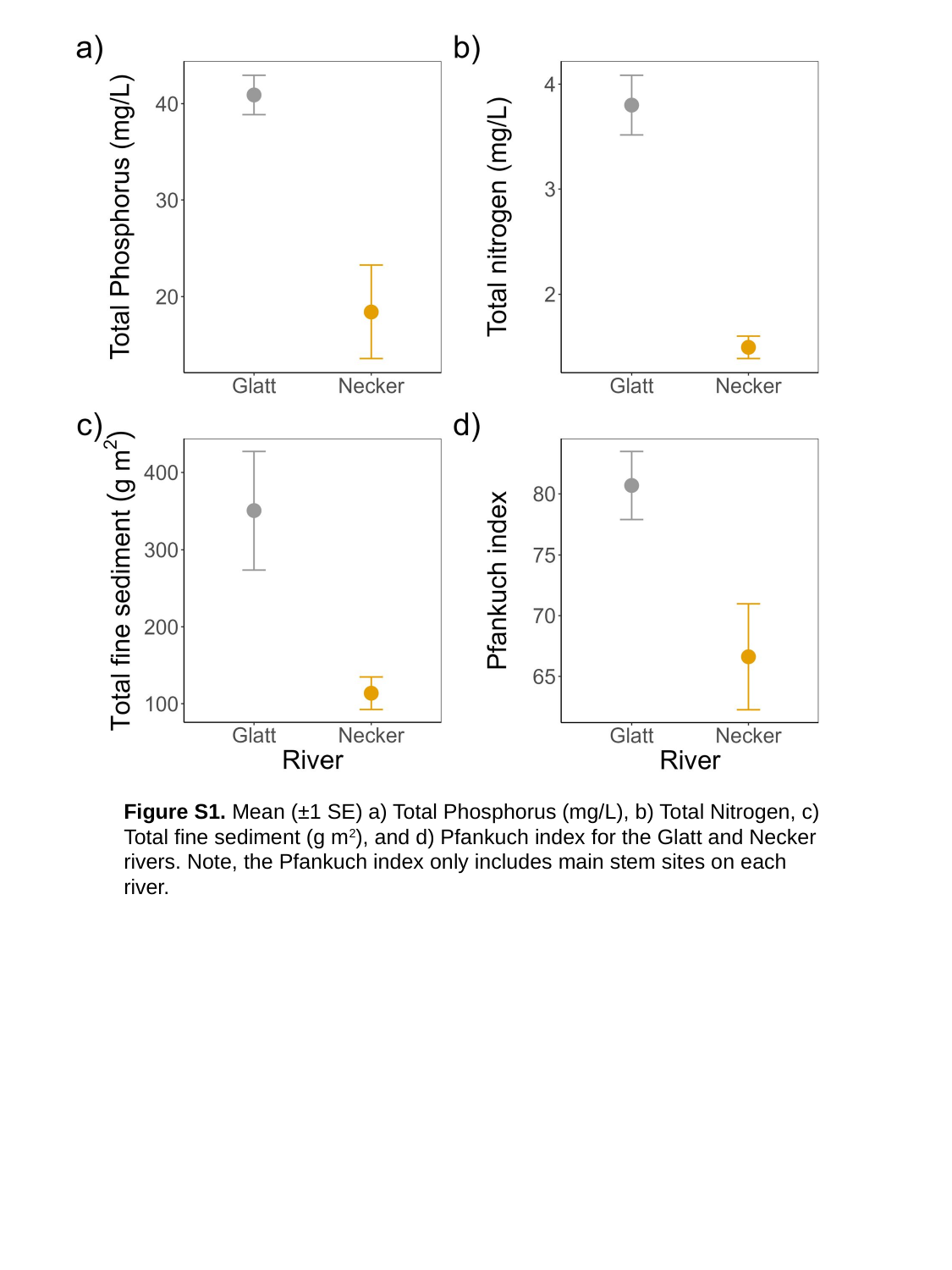

Figure S1. Mean (±1 SE) a) Total Phosphorus (mg/L), b) Total Nitrogen, c) Total fine sediment (g m2), and d) Pfankuch index for the Glatt and Necker rivers. Note, the Pfankuch index only includes main stem sites on each river.

## Slide 3
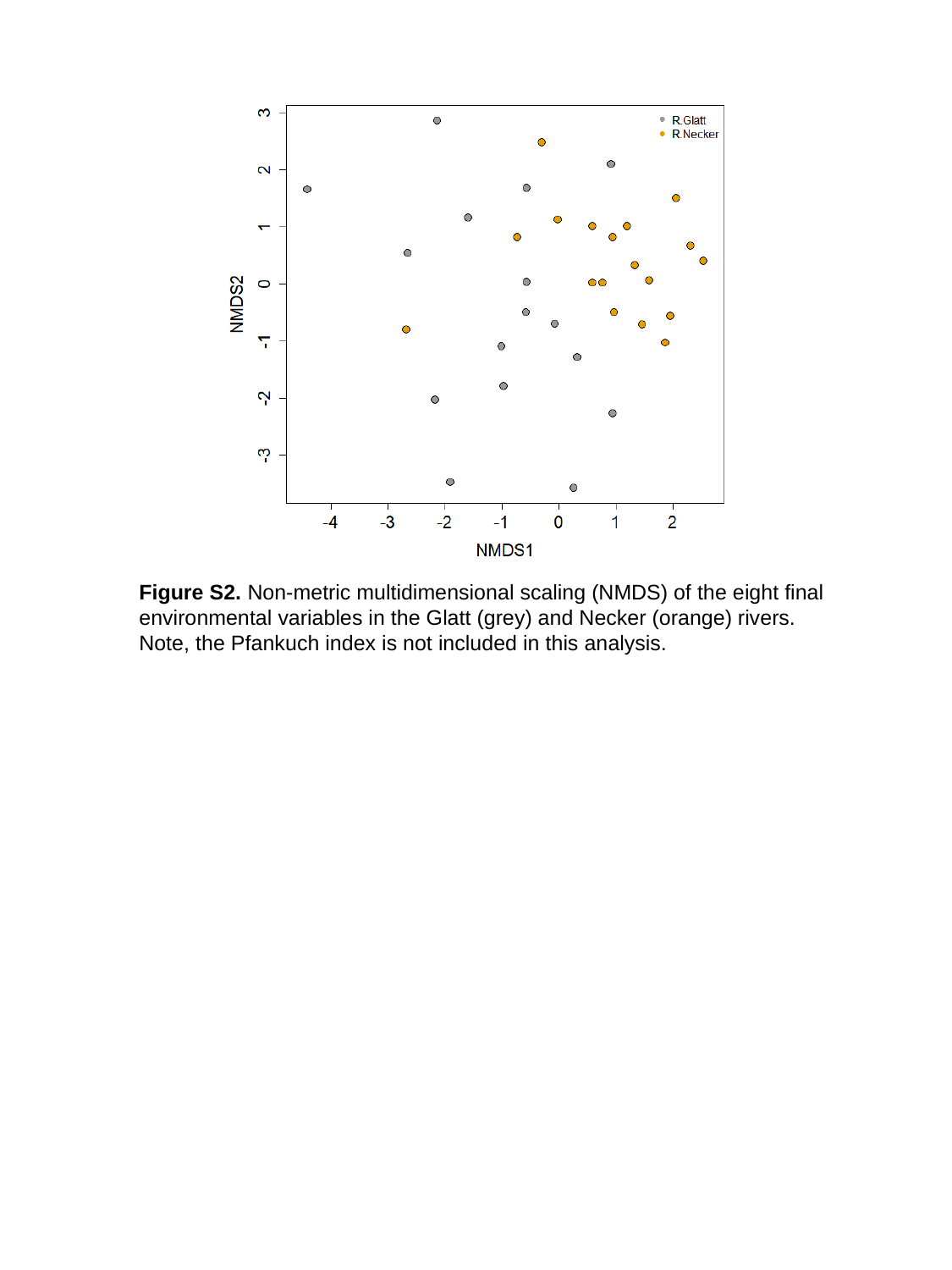

Figure S2. Non-metric multidimensional scaling (NMDS) of the eight final environmental variables in the Glatt (grey) and Necker (orange) rivers. Note, the Pfankuch index is not included in this analysis.

## Slide 4
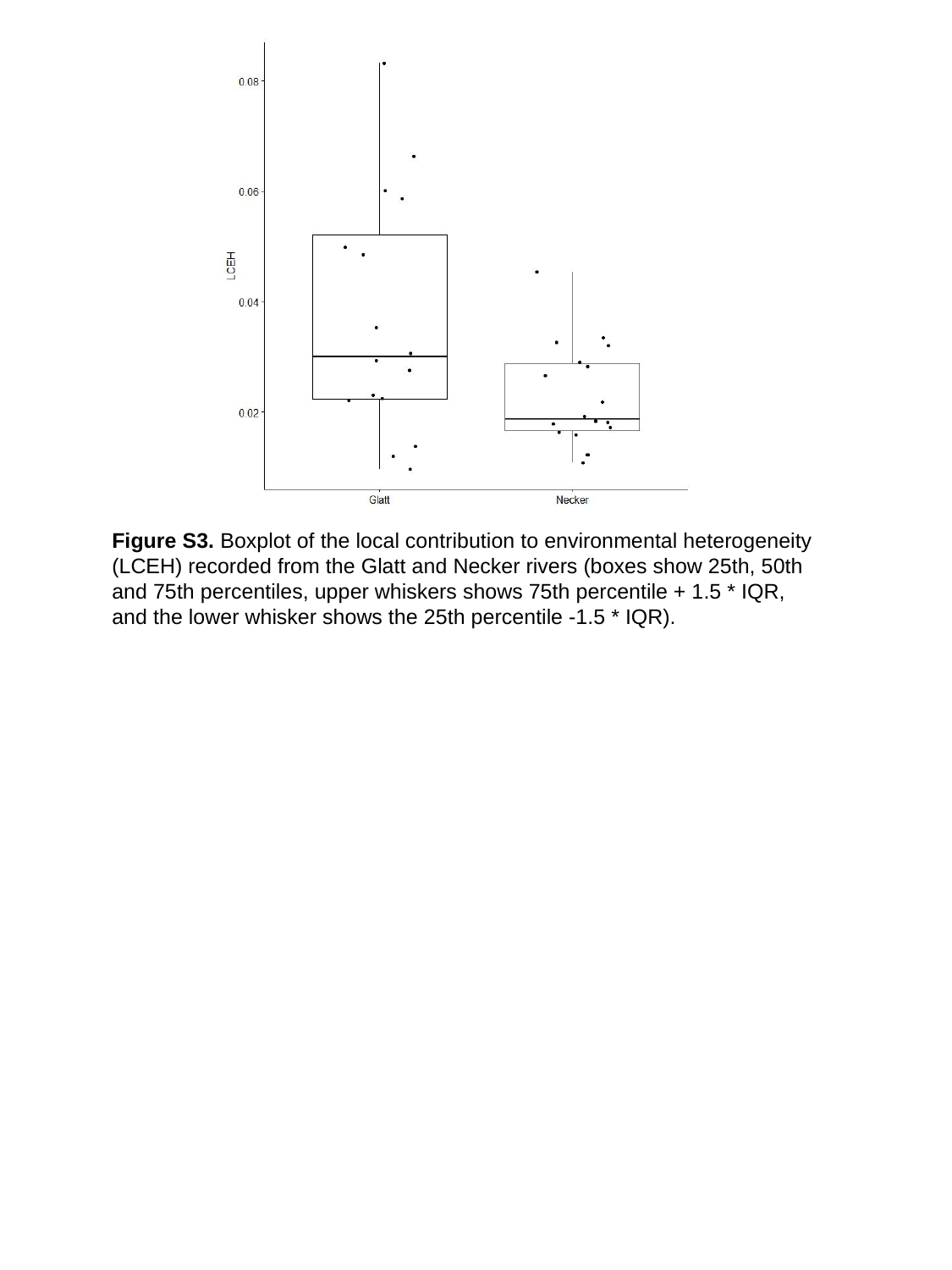

Figure S3. Boxplot of the local contribution to environmental heterogeneity (LCEH) recorded from the Glatt and Necker rivers (boxes show 25th, 50th and 75th percentiles, upper whiskers shows 75th percentile + 1.5 * IQR, and the lower whisker shows the 25th percentile -1.5 * IQR).

## Slide 5
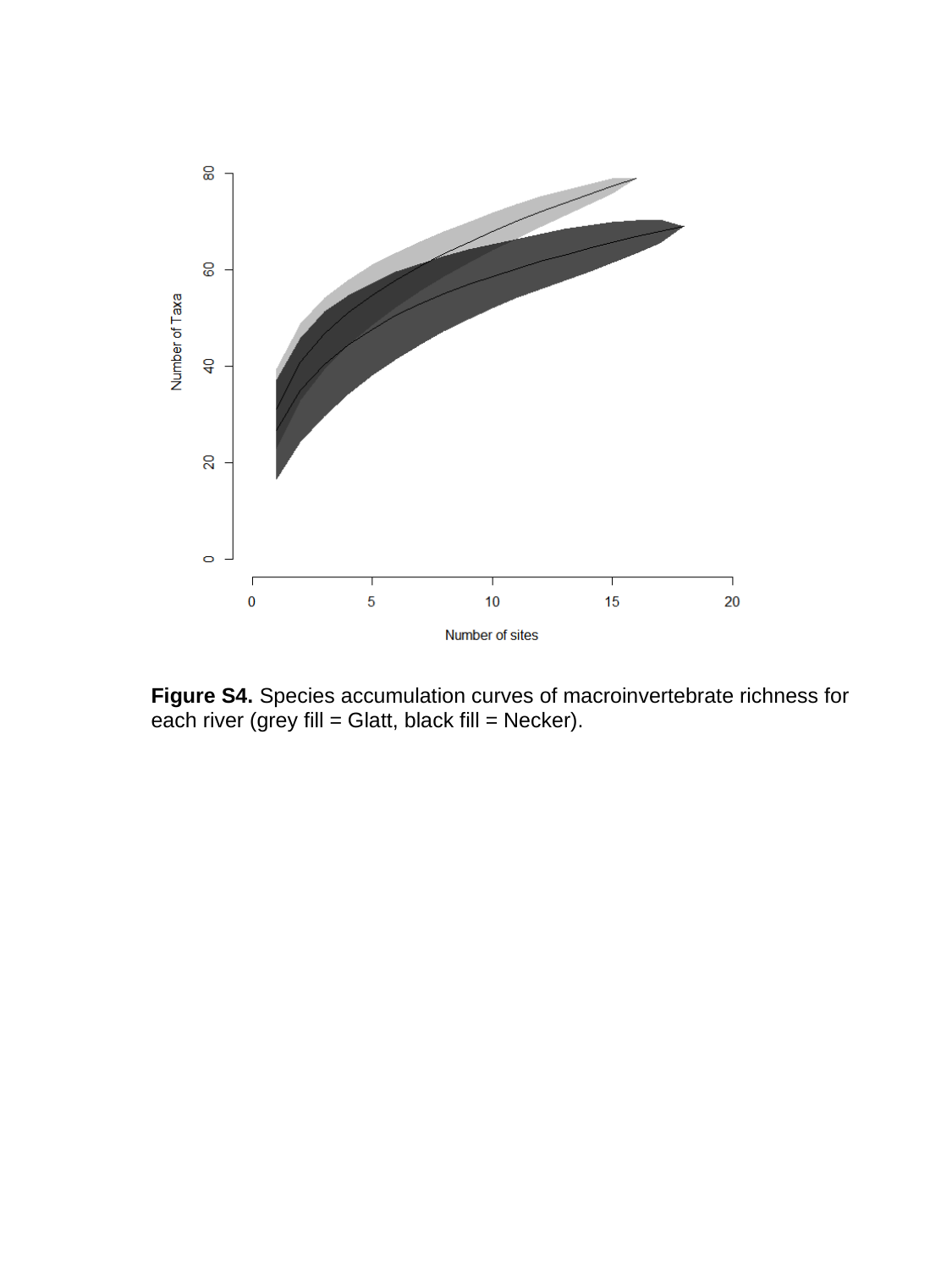

Figure S4. Species accumulation curves of macroinvertebrate richness for each river (grey fill = Glatt, black fill = Necker).

## Slide 6
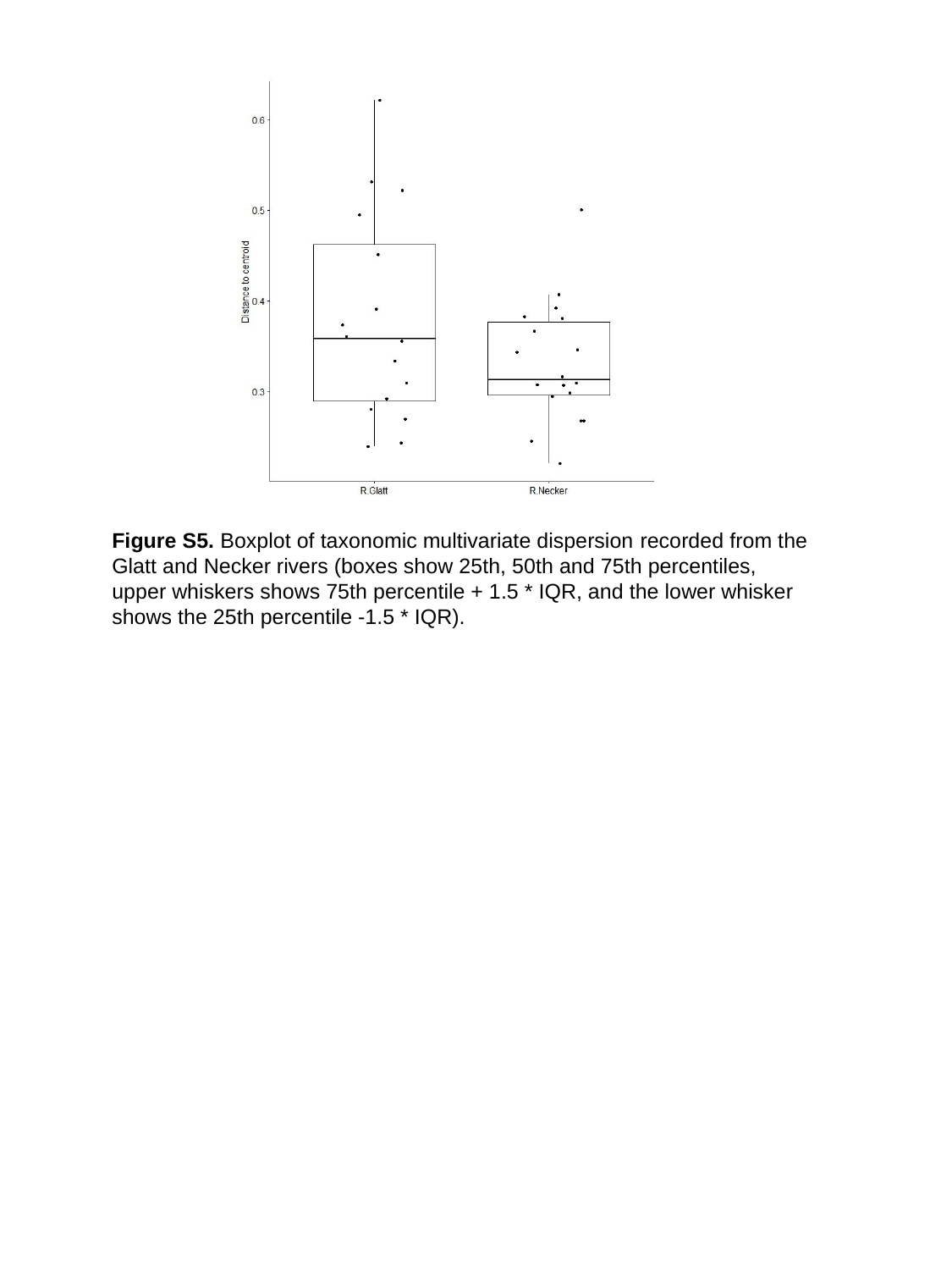

Figure S5. Boxplot of taxonomic multivariate dispersion recorded from the Glatt and Necker rivers (boxes show 25th, 50th and 75th percentiles, upper whiskers shows 75th percentile + 1.5 * IQR, and the lower whisker shows the 25th percentile -1.5 * IQR).

## Slide 7
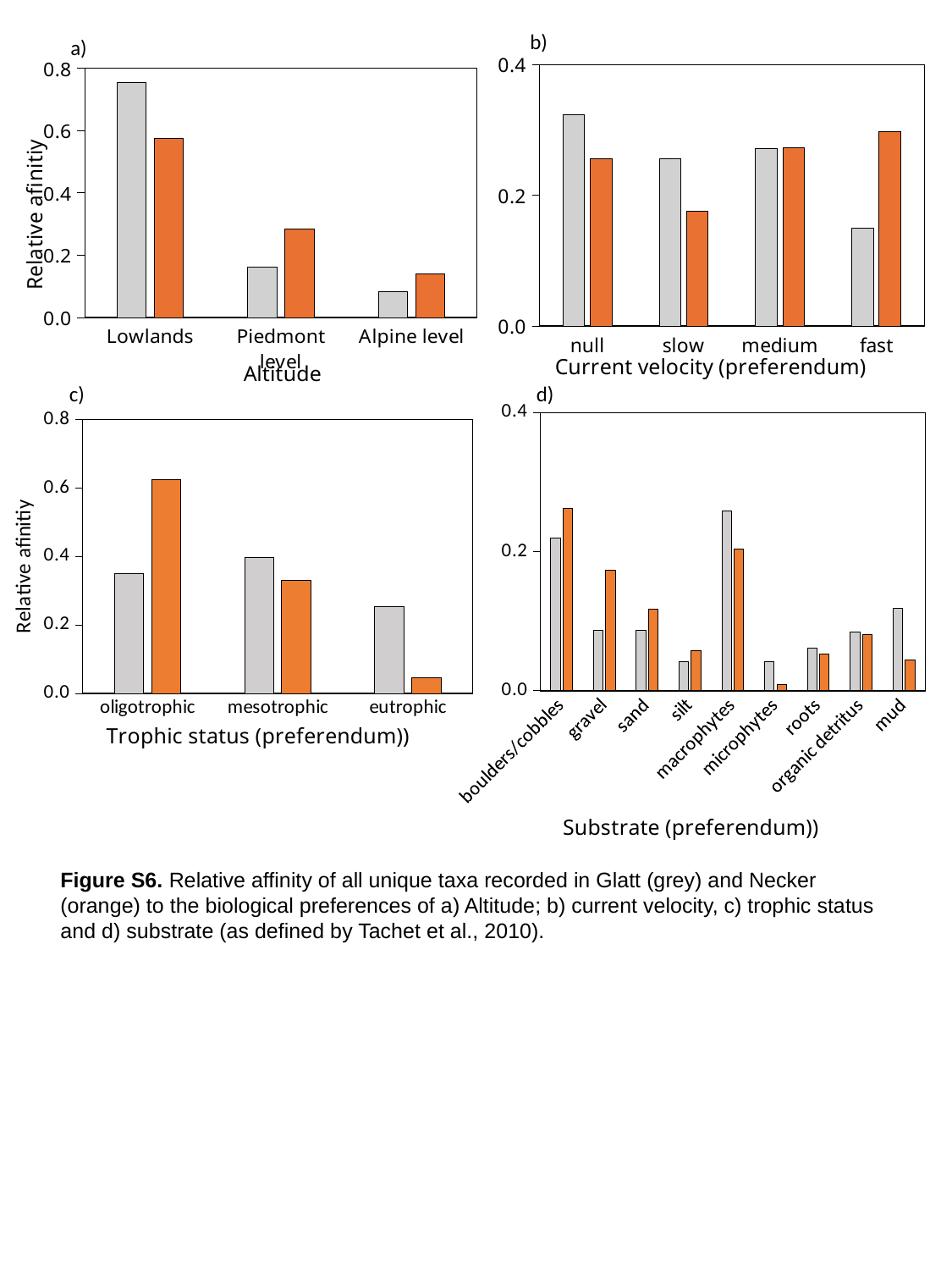

b)
a)
### Chart
| Category | Glatt | Necker |
|---|---|---|
| null | 0.3226190476190477 | 0.2555555555555556 |
| slow | 0.2555357142857143 | 0.17499999999999996 |
| medium | 0.2718452380952381 | 0.2722222222222222 |
| fast | 0.15 | 0.2972222222222222 |
### Chart
| Category | Glatt | Necker |
|---|---|---|
| Lowlands | 0.7543772893772893 | 0.5747252747252747 |
| Piedmont level | 0.16332417582417583 | 0.2838827838827838 |
| Alpine level | 0.08229853479853479 | 0.14139194139194136 |c)
d)
### Chart
| Category | Glatt | 0.63 |
|---|---|---|
| oligotrophic | 0.34922600619195043 | 0.6256410256410256 |
| mesotrophic | 0.39798761609907113 | 0.3294871794871795 |
| eutrophic | 0.2527863777089784 | 0.044871794871794865 |
### Chart
| Category | Glatt | Necker |
|---|---|---|
| boulders/cobbles | 0.2196187925504814 | 0.2620170176731263 |
| gravel | 0.08725137870773544 | 0.17287589233290593 |
| sand | 0.08714424095828269 | 0.11743133688835046 |
| silt | 0.04154031505075149 | 0.05753070458952812 |
| macrophytes | 0.2587258635313664 | 0.2039884449386712 |
| microphytes | 0.04130549189372719 | 0.008547008547008546 |
| roots | 0.06180987945219444 | 0.052554346106382314 |
| organic detritus | 0.08417490268533911 | 0.08049768230763704 |
| mud | 0.11842913517012188 | 0.04455756661639014 |Figure S6. Relative affinity of all unique taxa recorded in Glatt (grey) and Necker (orange) to the biological preferences of a) Altitude; b) current velocity, c) trophic status and d) substrate (as defined by Tachet et al., 2010).
